# RAS testing in metastatic colorectal cancer: excellent reproducibility amongst 17 Dutch pathology centers

**DOI:** 10.18632/oncotarget.3804

**Published:** 2015-04-12

**Authors:** Annemarie Boleij, Bastiaan B.J. Tops, Paul D.M. Rombout, Elizabeth M. Dequeker, Marjolijn J.L. Ligtenberg, J. Han van Krieken, Dutch RAS EQA Initiative

**Affiliations:** ^1^ Department of Pathology, Radboud University Medical Center, Nijmegen, The Netherlands; ^2^ Department of Human Genetics, Radboud University Medical Center, Nijmegen, The Netherlands; ^3^ Department of Public Health and Primary Care, Biomedical Quality Assurance Research Unit, KU Leuven - University of Leuven, Leuven, Belgium; ^4^ C.J.M. van Noesel, Academic Medical Center (AMC), Department of Pathology, Amsterdam; ^5^ C.C. Scheidel-Jacobse (Technical specialist), J.A. Kummer (Pathologist/KMBP), P. Roepman (KMBP in training), St. Antonius Ziekenhuis, Department of Pathology, Nieuwegein; ^6^ C.F.M. Prinsen (KMBP), S.H.M. van den Berg-van Erp (Pathologist), Canisius Wilhelmina Ziekenhuis (CWZ), Department of Pathology, Nijmegen; ^7^ J.M.H.H. van Gorp, Diakonessenhuis, Laboratory for Pathology, Utrecht; ^8^ P.M. Nederlof, Dutch Cancer Institute (NKI), Amsterdam; ^9^ E. Caspers, St. Elisabeth Ziekenhuis, Department of Molecular Pathology, Tilburg; ^10^ W.N.M. Dinjens, E.C.W. Beerens, Erasmus MC, Department of Pathology, Molecular Diagnostics, Rotterdam; ^11^ N.A. ‘t Hart, Isala, Department of Pathology, Zwolle; ^12^ A.J.C. van den Brule, Jeroen Bosch Ziekenhuis, Molecular Diagnostics, ‘s-Hertogenbosch; ^13^ R. van der Geize (KMBP), S.A. Riemersma (Pathologist), Laboratory for Pathology Oost-Nederland (LABPON), Hengelo; ^14^ T. van Wezel (KMBP), H. Morreau (Pathologist), R. van Eijk (Technical specialist), Leiden University Medical Center (LUMC), Department of Pathology, Leiden; ^15^ J.W.M. Jeuken, Laboratory for Pathology and Medical Microbiology (PAMM), Eindhoven; ^16^ A. Dirkx, Pathan B.V., Molecular Diagnostics, Rotterdam; ^17^ M. Klomp, Rijnstate Ziekenhuis, Department of Pathology, Arnhem; ^18^ W.T.M van Blokland, University Medical Center (UMC) Utrecht, Molecular Pathology, Utrecht; ^19^ A. ter Elst (Technical specialist/KMBP in training), E. Schuuring (KMBP), A. Diepstra (Pathologist), University Medical Center Groningen (UMCG), Department of Pathology, Groningen; ^20^ D.A.M. Heideman (KMBP), N.C.T. van Grieken (Pathologist), D. Sie (Technical specialist), VU-University Medical Center (VUMC), Department of Pathology, Amsterdam

**Keywords:** RAS, colorectal cancer, metastasis, quality control, next generation sequencing

## Abstract

In 2013 the European Medicine Agency (EMA) restricted the indication for anti-EGFR targeted therapy to metastatic colorectal cancer (mCRC) with a wild-type *RAS* gene, increasing the need for reliable *RAS* mutation testing. We evaluated the completeness and reproducibility of *RAS*-testing in the Netherlands.

From 17 laboratories, tumor DNA of the first 10 CRC cases tested in 2014 in routine clinical practice was re-tested by a reference laboratory using a custom next generation sequencing panel. In total, 171 CRC cases were re-evaluated for hotspot mutations in *KRAS*, *NRAS* and *BRAF*.

Most laboratories had introduced complete *RAS*-testing (65%) and *BRAF*-testing (71%) by January 2014. The most employed method for all hotspot regions was Sanger sequencing (range 35.7 – 49.2%). The reference laboratory detected all mutations that had been found in the participating laboratories (n = 92), plus 10 additional mutations. This concerned three *RAS* and seven *BRAF* mutations that were missed due to incomplete testing of the participating laboratory. Overall, the concordance of tests performed by both the reference and participating laboratory was 100% (163/163; κ-static 1.0) for *RAS* and 100% (144/144; κ-static 1.0) for *BRAF*.

Our study shows that *RAS* and *BRAF* mutations can be reproducibly assessed using a variety of testing methods.

## INTRODUCTION

Anti-Epithelial Growth Factor Receptor (EGFR) targeted therapy, such as panitumumab and cetuximab, is effectively reducing the risk of tumor progression and improving overall survival (OS), progression free survival (PFS) and quality of life in metastatic colorectal cancer (mCRC) patients whose tumor is *RAS* wild-type [1, 2]. Conversely, mCRC patients with mutated *RAS* tumors (*KRAS* and *NRAS* mutations) who received panitumumab in combination with oxaliplatin-based chemotherapy had a significantly worse outcome in OS and PFS [3]. This notion has led to a new incentive by the European Medicine Agency (EMA) for Vectibix (panitumumab) in June 2013 [4] and for Erbitux (cetuximab) in November 2013 [5], to indicate administration of EGFR targeted therapy only to patients with wild-type *RAS* mCRC. This has increased the need for reliable *RAS* mutation testing methods to assure the quality of *RAS* status determination.

Most molecular testing methods that are used nowadays accurately assess mutational status of *RAS* genes in samples with >30-50% tumor cells, or alternatively with 15-25% of mutated alleles in the test sample [6, 7]. However, with lower number of mutated alleles in the sample, the limit of accurate detection of a method declines depending on the test method used [8]. Even when using the same method, differences in protocols between laboratories can result in different outcomes. It has been suggested that the reproducibility amongst different testing methods is not as high as anticipated for based on previous EQA schemes for *KRAS* exon 2 testing [9]. In a recent study, in 29 out of 182 *KRAS* exon 2 wild-type tumors (15.9%), as assessed with Sanger sequencing, a *KRAS* exon 2 mutation was found with next generation sequencing (NGS) [10]. This suggests a higher variability in reproducibility between test methods and laboratories than initially measured [11].

Given the clinical impact of *RAS*-testing, it is of utmost importance to control for reliable performance of routine *RAS*-testing methods used in clinical practice. In this study, we evaluated the inter-laboratory agreement of *RAS*-testing amongst 171 mCRC patients of 17 Dutch laboratories.

## RESULTS

### Integration of full RAS-testing in the Netherlands

In January 2014, 11 of 17 participating Dutch laboratories had introduced full *RAS* testing (65%). All 17 participating centers performed *KRAS* exon 2 (codon 12 and 13) and *KRAS* exon 3 (codon 61) testing. The method most frequently used for *KRAS* exon 2 was Sanger sequencing of PCR products, either directly (5 laboratories; 51 samples (29.8%)) or to specify the mutation detected with high resolution melting (HRM) analysis or to confirm a real-time PCR result (7 laboratories; 70 samples (40.9%)) [Table 1].

**Table 1:** Methods used to test for *KRAS*, *NRAS* and *BRAF* mutations

	*KRAS*					*NRAS*					*BRAF*
Method*	codon *12/13* N (%)	codon 59 N(%)	codon 61 N (%)	codon 117 N (%)	codon 146 N(%)	codon 12/13 N (%)	codon 59 N (%)	codon 61 N(%)	codon 117 N (%)	codon 146 N (%)	codon 600 N (%)
**Next generation sequencing**											
Illumina MiSeq Oncopanet (Illumina)[25]	10 (5,8)	10 (5,8)	10 (5,8)	10 (5,8)	10 (5,8)	10 (5,8)	10 (5,8)	10 (5,8)	0 (0,0)	0 (0,0)	10 (5,8)
IonTorrent (Life Sciences)	20 (11,7)	20 (11,7)	20 (11,7)	20 (11,7)	20 (11,7)	20 (11,7)	20 (11,7)	20 (11,7)	10 (5,8)	20 (11,7)	20 (11,7)
**Sequencing**											
Sanger Sequencing	51 (29,8)	**61** **(35,7)**	**61** **(35,7)**	**73** **(42,7)**	**84** **(19,2)**	**74** **(43,3)**	**84** **(49,2)**	**74** **(434)**	**83** **(48,6)**	**73** **(487)**	**51** **(29,8)**
HRM + Sanger Sequencing	**60** **(35.1)**	60 (15.1)	60 (35.1)	30 (17.5)	30 (17.5)	.30 117.5)	30 (17.5)	30 (17.5)	0 (0.0)	0 (0.0)	40 (23.4)
Real-time PCR + Sanger sequencing	10 (5,8)	0 (0,0)	0 (0,0)	0 (0,0)	0 (0,0)	10 (5,8)	0 (0,0)	10 (5,8)	0 (0,0)	0 (0,0)	10 (5,8)
Pyrosequencing	0 (0,0)	0 (0,0)	0 (0,0)	0 (0,0)	0 (0,0)	0 (0,0)	0 (0,0)	0 (0,0)	10 (5,8)	10 (5,8)	0 (0,0)
**Other assays**											
Therascreen KRAS/NRAS/*BRAF* Pyrokit (Qiagen)	10 (5,8)	0 (0,0)	10 (5,8)	0 (0,0)	0 (0,0)	10 (5,8)	0 (0,0)	10 (5,8)	0 (0,0)	0 (0,0)	10 (5,8)
Therascreen *RAS* extension Pyrokit (Qiagen)	0 (0,0)	10 (5,8)	0 (0,0)	10 (5,8)	10 (5,8)	0 (0,0)	10 (5,8)	0 (0,0)	10 (5,8)	10 (5,8)	-
Sequenom MassArray (Sequenom)	10 (5,8)	0 (0,0)	10 (5,8)	10 (5,8)	10 (5,8)	10 (5,8)	10 (5,8)	10 (5,8)	10 (5,8)	10 (5,8)	10 (5,8)
**Total samples tested**	**171** **(100)**	**161** **(94,2)**	**171** **(100)**	**153** **(89,5)**	**161** **(95,9)**	**164** **(95,9)**	**164** **(95,9)**	**161** **(95,9)**	**123** **(71,9)**	**123** **(71,9)**	**151** (88,3)*

* Total 171 samples received from 17 labs, 10 samples per lab, 1 lab sent 11 DNA samples;

* 4 laboratories only *test BRAF* on request by the physician.

Note: for each codon. the most frequently used method is highlighted in bold

Of the 6 laboratories that had not introduced full *RAS*-testing, one laboratory did not test for *NRAS* exon 2, 3, 4 and *KRAS* exon 4, three had not introduced *NRAS* exon 4 (codon 117 and 146), one laboratory did not have *KRAS* exon 4 codon 117 in their test panel and one laboratory did not test for KRAS exon 3 codon 59 [see Table 1]. One of these 6 laboratories introduced full *RAS* testing early 2014 and had only tested 3 out of 10 (30%) CRC cases with full *RAS*-testing.

For full *RAS*-testing direct Sanger sequencing of PCR products was used most often either with or without a prescreen (range 35.7% – 49.2%) [Table 1]. The use of Mass Spectrometry (Sequenom) or CE-IVD kits (Therascreen) was reported by 2 laboratories (11.6%). In 3 laboratories (17.5%) next generation sequencing (NGS) with MiSeq (Illumina) or Ion Torrent (Life technologies) was used for *RAS* testing. In conclusion, full *RAS* testing had been introduced in the majority of laboratories participating in this study and relied mostly on Sanger sequencing methods.

### Mutation frequencies of *KRAS*, *NRAS* and *BRAF*

**Figure 1:**
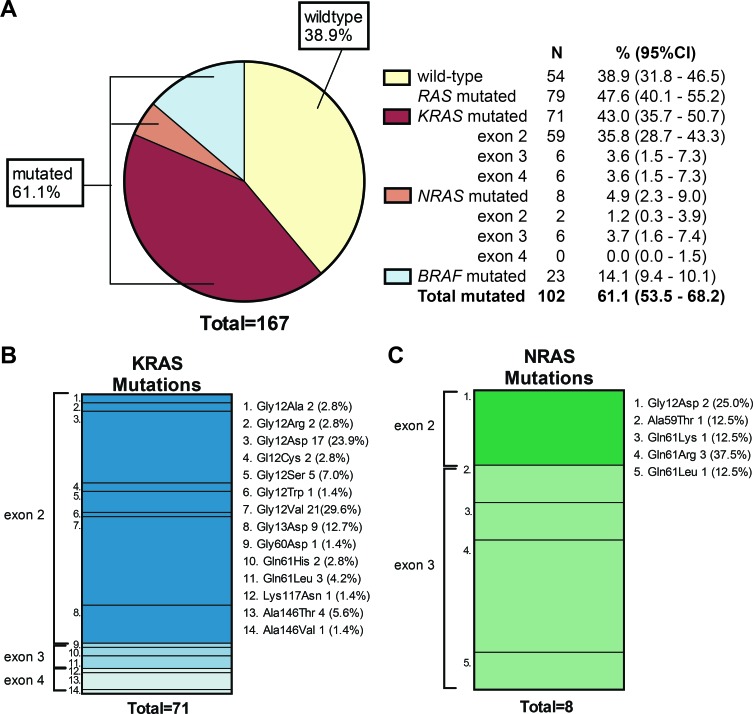
Mutation prevalence of *KRAS*, *NRAS* and *BRAF* **A**) Pie chart showing all evaluated CRC cases (*n* = 167; 167 of 171 samples could be evaluated for at least one of the target sites). Of these CRC cases, 61.1% had a mutation. *KRAS* mutations alone were most frequently observed (43.0%). *BRAF* mutations were found in 23 CRC cases (All in exon 15; c.1799T>A (p.Val600Glu)). The 95% CI was calculated with Jeffry's method. **B**) A total of 71 *KRAS* mutations was found; 59 in *KRAS* exon 2 (83.2%), 6 in *KRAS* exon 3 (8.4%), and 6 in *KRAS* exon 4 (8.4%). Gly12Val (29.6%) and Gly12Asp (23.9%) were the most common mutations found followed by Gly13Asp (12.7%). **C**) A total of 8 NRAS mutations was detected of which 2 were detected in exon 2 (Gly12Asp) and 6 in exon 3. The most common mutation in *NRAS* was Gln61Arg (37.5%).

The amount and quality of 167 out of 171 received DNA samples was sufficient for successful evaluation by the reference laboratory using NGS of at least one of the target sites (165 samples for *KRAS,* 163 for *NRAS* and 163 for *BRAF*; see materials and methods). Overall, in 102 samples (61.1% (95% CI 53.5 – 68.2)) a mutation in *KRAS*, *NRAS* or *BRAF* was found at the reference laboratory [Figure 1A].

A *KRAS* exon 2 mutation was detected in 59 mCRC cases (35.8%), other *RAS* mutations were found in *KRAS* exon 3 (*n* = 3 (3.6%)), *KRAS* exon 4 (*n* = 3 (3.6%)) *NRAS* exon 2 (*n* = 1 (1.2%)) and *NRAS* exon 3 (*n* = 3 (3.6%)). No mutations in *NRAS* exon 4 were detected. This resulted in a total 79 *RAS* mutations (47.6% (95% CI 40.1-55.2)) of which only 8 were *NRAS* mutations. The mean percentage of mutated alleles was 42.1% (SD 15.7%) for *KRAS* and 46.1% (SD 23.25%) for *NRAS* [Supplementary Table 1]. The majority of *KRAS* mutations affected codon 12 (70.5%), especially p.Gly12Asp (23.9%) and p.Gly12Val were common (29.6%) [Figure 1B]; 5 out of 8 *NRAS* mutations were found in codon 61 (62.5%) [Figure 1C]. None of the samples harbored both or more than one *KRAS* and/or *NRAS* mutation.

*BRAF* mutations occurred in 23 of 163 CRC cases (14.1% (95%CI 9.4-20.1)) [Figure 1A] and were all c.1799T>A (p.Val600Glu) mutations in exon 15. The mean percentage of mutated alleles for *BRAF* was 34.5% (SD 14.3%) [Supplementary Table 1]. Both *KRAS* and *NRAS* mutations in our sample were mutually exclusive with *BRAF* mutations (OR =2.15 (95%CI 1.80-2.57); Chi-square p<0.01).

### High concordance of *RAS* and *BRAF*-testing

For 167 samples that could be evaluated by the reference laboratory, all mutations that had been found in the participating laboratories were verified. In addition, the reference lab detected three *RAS* and seven *BRAF* mutations that had not been detected in the participating laboratories. This concerned mutations in *KRAS* exon 4 (c.436G>A; p.Ala146Thr), *NRAS* exon 2 (c.35G>A; p.Gly12Asp), *NRAS* exon 3 (c.181C>A; p.Gln61Lys) and seven mutations in *BRAF* (c.1799T>A; p.Val600GLu) that the participating laboratory had not tested for [Table 2]. Altogether, the inter-laboratory agreement for tests performed by both the reference and participating laboratory was 100% (163/163) (κ statistic 1.0) for full *RAS* and 100% (144/144) (κ statistic 1.0) for *BRAF*-testing [Table 3]. All three additionally found *RAS*-mutations were reported back to the respective participating laboratory, and were confirmed with their newly installed Sanger sequencing or NGS approaches for the respective target sites.

**Table 2:** Additional mutations found by the reference laboratory

Gene	Exon	N samples	Amino acid change	Reason not found by participating laboratory
*KRAS*	4	1	p.Alal46Thr	not in test repertoire
*NRAS*	2	1	p.Glyl2Asp	not in test repertoire
*NRAS*	3	1	p.Gln6lLys	not in test repertoire
*BRAF*	15	4	p.Val600Glu	*BRAF* tested only on request by physician
*BRAF*	15	3	p.Val600Glu	not in test repertoire

**Table 3:** Inter-laboratory agreement between participating and reference laboratory

	Samples	N(%)^‡^	Wildtype		Mutated		Inter-laboratory agreement^†^	
			participating laboratory	reference laboratory	participating laboratory	reference laboratory	concordance	k-statistic
***RAS***	163*	**(95,3)**	**87**	**87**	**76**	**76**	**100%**	**1,0**
*KRAS*	*164*	*(95,9)*	*94*	*94*	*70*	*70*	*100%*	*1,0*
*exon 2*	*165*	*(96,5)*	*106*	*106*	*59*	*59*	*100%*	*1,0*
*exon 3*	*165*	*(96,5)*	*159*	*159*	*6*	*6*	*100%*	*1,0*
*exon 4*	*159*	*(93,0)*	*154*	*154*	*5*	*5*	*100%*	*1,0*
*NRAS*	*157*	*(91,8)*	*151*	*151*	*6*	*6*	*100%*	*1,0*
*exon 2*	*157*	*(91,8)*	*156*	*156*	*1*	*1*	*100%*	*1,0*
*exon 3*	*157*	*(91,8)*	*152*	*152*	*5*	*5*	*100%*	*1,0*
*exon 4*	*117*	*(68,4)*	*117*	*117*	*0*	*0*	*100%*	*1,0*
***BRAF***	144**	(84,2)	128	128	16	16	100%	1,0

† wild-type and mutated as found by the reference laboratory

‡ Samples that were not included could not be evaluated due to low DNA concentration or low read coverage, or were not tested by both reference laboratory and the participating laboratory

* in 163 of the 171 samples at least 1 of the target exons could be evaluated in the reference laboratory that was also tested by the participating laboratory. The following samples that were tested by the participating laboratory could not be evaluated in the reference laboratory due to low read coverage as result of low or poor DNA quality: *KRAS* exon 2: 6 of the 171 samples tested; *KRAS* exon 3: 6 of the 171 samples tested; *KRAS* exon 4; 5 of the 164 samples tested; *NRAS* exon 2; 7 of the.164 samples tested; *NRAS* exon 3; 7 of the 164. samples tested; *NRAS* exon 4; 6 of the 123 samples tested.

** 7 of the 151 samples tested for *BRAF* by the participating laboratory could not be evaluates in the reference laboratory due to low read coverage as result of low or poor DNA quality.

### Association between percentage of neoplastic cells and mutation detection

**Figure 2:**
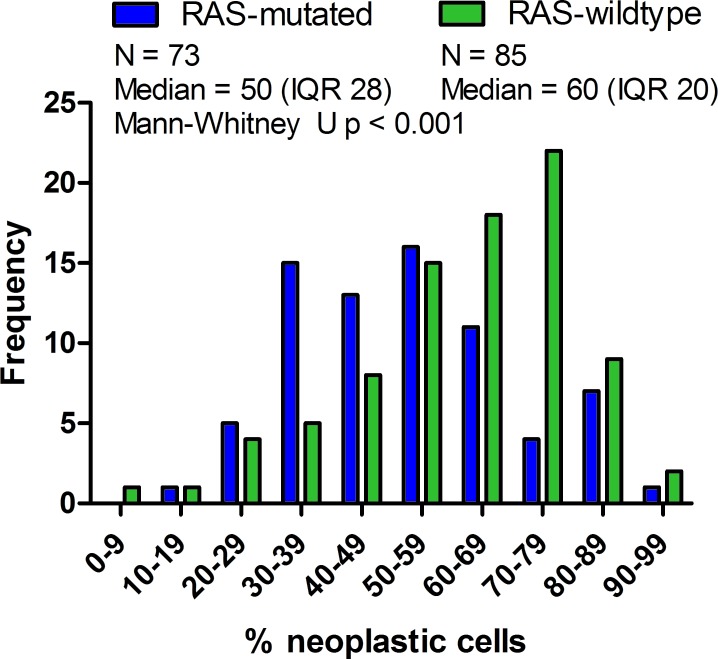
Neoplastic cell percentage in *RAS*-mutated and *RAS*-wild-type tumors The distribution of the estimated percentage of tumor cells in the test sample was compared between *RAS*-mutated and *RAS*-wild type CRC cases. Frequency of CRC cases is shown on the Y-axis; the X-axis represents the percentage of neoplastic cells. Mann-Whitney U test of the comparison indicates that the median neoplastic cell percentage of *RAS*-mutated mCRC cases is significantly lower (Median 50 (IQR 28), Mean Rank = 66,65) than RAS-wild-type mCRC cases (Median 60 (IQR 20), Mean Rank = 90,54; *p* = 0.001) and thus that the populations have distinct neoplastic cell percentage distributions.

Testing sensitivity of *RAS*-mutations is dependent on the percentage of neoplastic cells represented in the test sample; low neoplastic cell percentages may result in missing of *RAS*-mutations depending on the technique that is used [8]. For 158 samples the percentage of neoplastic cells as estimated by the pathologist of the participating laboratory was known. The median estimated neoplastic cell percentage represented in the DNA samples was 50% (inter quartile range (IQR)=30). Of the 158 samples, 47 (29.7%) had neoplastic cell percentages below 40%. When comparing the distribution of estimated neoplastic cell percentages amongst *RAS*-mutated (*N* = 73) and *RAS*-wild-type (*N* = 85) samples, the median neoplastic cell percentage was significantly lower in *RAS*-mutated samples (50.0% (IQR=28) compared to *RAS*-wild type (60.0% (IQR=20)) samples (Mann-Whitney U, *p* < 0.01) [Figure 2]. In *RAS*-mutated samples, the mean percentage of mutated alleles correlated with the percentage of neoplastic cells in the test sample (Pearson r = 0.433 (*p* < 0.01) for *KRAS* and Pearson *r* = 0.792 (*p* = 0.034) for *NRAS*; Supplementary Figure 1).

The frequency of *RAS* mutations amongst samples with neoplastic cell percentages ≤40% was significantly higher than in samples >40% neoplastic cells (OR 2.45 (95%CI 1.22 – 4.94), chi-square *p* = 0.011). A similar result was obtained when lowering the cut-off to 30 or 20% neoplastic cells, suggesting that the sensitivity of the *RAS* mutation analyses were adequate, even in samples with low neoplastic cells. However, care should be taken while reporting wild-type in samples with low neoplastic cells depending on the lower limit of detection of the technique applied to prevent reporting false-negatives.

## DISCUSSION

Our results show an excellent concordance (100%, kappa 1.0) of *RAS*-test results of the reference lab oratory and 17 other Dutch laboratories in routine clinical practice, despite differences in testing methods used. Overall, full *RAS*-testing of *KRAS* exon 2, 3 and 4 and *NRAS* exon 2, 3 and 4 has been introduced in the majority of the participating laboratories (65%). Three *RAS* mutations and seven *BRAF* mutation were missed due to incomplete testing.

The frequency of *RAS* mutations reported in our study matches those previously reported in the literature. Approximately 35-45% of all CRCs contain *RAS*-mutations in *KRAS* exon 2 [3, 12-16], whereas approximately 10% of *RAS*-mutations occurs in *KRAS* exons 3 and 4 or *NRAS* exons 2, 3 and 4 [3, 17-19]. In addition, *KRAS*, *NRAS* and *BRAF* mutations have a strong tendency towards mutual exclusivity [20]. *BRAF* mutations occurred in 14% of our samples, which seems higher than the reported 9% in the Cancer Genome Atlas [20]. While *BRAF* mutation analysis is incorporated in the test repertoire of most laboratories (71%), some laboratories only test for *BRAF* on special request by the physician when tumors were previously tested *RAS* wild-type. *BRAF* mutation frequency amongst *KRAS* exon 2 wild-type tumors is reported to be around 8-15% [10, 18].

To assure accurate determination of *RAS* wild-type and mutant status, the quality of mutation detection for each *KRAS* and *NRAS* exon needs to be determined. Reproducibility is one of the measures that signify the quality of diagnostic tests. Poor reproducibility can have several causes: 1) the testing methods used have a difference in sensitivity (ability to identify tumors with the mutation) or, 2) there is variation between different persons/laboratories performing the test (inter-observer variability). In a recently published Italian study, it was found that amongst *KRAS* exon 2 wild-type tumors, as assessed with Sanger sequencing, a *KRAS* exon 2 mutation was found with NGS in 28 out of 182 mCRC cases (15.9%). The difference in sensitivity between the testing methods could have partly accounted for the discrepancy found in this study [10]. In our study the discrepancy between the test results generated in 17 different laboratories using a variety of testing methods and the reference laboratory using a NGS approach was naught, even in samples with a neoplastic cell percentage between 20-40% and in laboratories using Sanger sequencing. In the 2012 *KRAS* external quality assessment program of the European Society of Pathology, less than 5% of all samples were wrongly genotyped for *KRAS* amongst 100 laboratories in 26 countries [11].

Testing sensitivity is reflected by the limit of detection of the method, but is also limited by the percentage of neoplastic cells represented in the test sample. In fact, low neoplastic cell percentage (~10%) led to 16 of the 29 false-negatives in the 2012 *KRAS* EQA scheme [11]. For PCR and Sanger sequencing a minimum amount of 20-30% of tumor cells is required [21]. Our study has high reproducibility even in samples with a low neoplastic cell percentage and the percentage of mutated alleles correlates with the neoplastic cell content in the sample [Supplementary Figure 1]. In fact, the percentage of *RAS* mutant samples was significantly higher in samples with neoplastic cell percentages below 40%, which demonstrates that mutations could also be detected in samples with a low neoplastic cell percentage. However, this unexpected observation could be merely coincidental due to small subgroups and difference in tumor sampling in combination with variation in estimating the amount of neoplastic cells, which is known to be high among pathologists [22, 23].

Potential sources of bias in our study could have affected the reported reproducibility. First, centers were asked to send DNA of the first 10 CRC cases tested in 2014. Because *KRAS* exon 2 mutations are the most frequent, there were some laboratories where only *KRAS* exon 2 mutations or wild-type *RAS-*status was reported. This could have led to an overestimation of the reproducibility because other *RAS*-mutations were not present. Secondly, the likelihood of finding false-negatives is higher when only wild-type samples are re-tested. In this series only 54 samples were wild-type for all the tested hotspot mutations and thus could have led to an underestimation of false-negative wild-type samples. The small number of *NRAS*-mutations found has likely overestimated the reproducibility of this group of mutations. Future studies need to verify whether newly installed methods reproducibly detect *NRAS* mutations, especially in laboratories that did not report any *NRAS* mutations in the tested CRC panel.

In conclusion, our study clearly shows that *RAS*-status can be reproducibly assessed between laboratories in routine clinical practice using similar or different testing methods. With constant improvement of testing methodologies and quality controls, this offers good expectations for the future of molecular testing in mCRC. Nevertheless, more in depth analyses with regard to the effect of testing sensitivity and the percentage of tumor cells in the test sample on *RAS* mutation detection are warranted.

## PATIENTS AND METHODS

### Patient selection and data collection

All 22 Dutch institutes that participated in the European Quality Assurance (EQA) scheme of the European society of pathology (ESP) in 2013 were invited to participate in the study. Seventeen laboratories responded and were subsequently requested to send 10 μl DNA (≥30 ng) of the first 10 mCRCs tested in routine diagnostics for anti-EGFR targeted therapy from 1st January 2014. In addition, each laboratory was asked to indicate the testing method used, the mutations tested for and the percentage of neoplastic cells in the tissue the DNA was extracted from. Moreover, the *KRAS*, *NRAS* and *BRAF* mutations found by the participating laboratories were reported and stored by a third party until study end. Retesting of the samples at the reference laboratory was performed blinded. Approval by a medical ethics committee was not required. All data were reported in de-identified form and are in agreement with the Dutch Data Protection Act.

### Next generation sequencing

A total of 171 DNA samples received from the participating laboratories (one lab contributed 11 samples) were quantified on the Qubit platform (Life technologies). Samples with a DNA concentration below 0.5 ng/μl were excluded from further analysis. Bar-coded libraries were prepared from 10-100 ng DNA using a custom AmpliSeq panel targeting frequently mutated regions in the *KRAS, NRAS, EGFR, PIK3CA, ERBB2, AKT1, BRAF* genes and the *AMELX/Y* gene as a control gene to determine the sex of the patient. Libraries were equimolar pooled and clonal amplification was performed by emulsion PCR using the One Touch 2 system (Life Technologies) and subsequently run on the Ion Torrent Personal Genome Machine (Life Technologies). Torrent Suite Software v.3.4.2. was used to pre-process the raw data. Subsequent mapping and variant calling was performed using SeqNext software v.4.1.2. (JSI medical systems GmbH). The following genes and exons were included in the data analysis: *KRAS* exon 2, 3 and 4 (NM_004985.3), *NRAS* exon 2,3 and 4 (NM_002524.3) and *BRAF* exon 15 (NM_004333.4). The minimum read coverage allowed to call mutant alleles was set at 100 reads. In general, read coverage was more than 500 resulting in a sensitivity of 5% mutant alleles. For a read coverage between 100-500 reads, a limit of 10% mutant alleles was employed. When coverage was low or mutation calling could not be established due to other technical reasons, the run was repeated. When there was still insufficient coverage of the target sites after repeating the run, the samples were excluded from analysis. In total 6 *KRAS*, 8 *NRAS* and 8 *BRAF* target sites had insufficient coverage due to low amount or poor DNA quality. Therefore, we could successfully evaluate a total of 165 *KRAS*, 163 *NRAS* and 163 *BRAF* target sites; for 167 samples at least one of the target sites could be successfully evaluated.

### Data analysis

Variants were filtered for known SNPs and systematic sequencing artifacts. Next, the somatic mutations found in the DNA-samples by the reference laboratory were compared with the mutations originally found by the participating laboratories to assess the percentage of agreement of the results. The mutations were compared at the genotype level. Each participating pathology center was informed about their individual results and performance.

### Statistical analysis

All statistical analyses were performed in IBM SPSS statistics version 20. Mutation frequencies were calculated for *KRAS*, *NRAS* and *BRAF*. Binominal confidence intervals were calculated using the Jeffrey's interval. The detected mutation(s) in the DNA-samples at the genotype level were compared between the reference laboratory and the participating laboratories and expressed as percentage agreement. Data were evaluated for hotspot mutations in *KRAS* exon 2, 3 and 4, *NRAS* exon 2, 3 and 4 and *BRAF* exon 15. Concordance (inter-laboratory agreement, kappa-statistic [24]) was only calculated when the mutation was targeted in the test panel of both the reference and participating laboratory. Odds ratios with 95% confidence intervals and chi-square statistics were calculated to evaluate associations between *RAS*-mutations and *BRAF*. The distribution of tumor cell percentage in the DNA-samples amongst *RAS*-mutated and *RAS*-wild-type samples was assessed with the Mann-Whitney U test.

## SUPPLEMENTARY MATERIAL FIGURE AND TABLE




