# Supplementary material for: RAS testing in metastatic colorectal cancer: excellent reproducibility amongst 17 Dutch pathology centers
Source: Oncotarget. 2015 Apr 12;6(17):15681–9. doi: 10.18632/oncotarget.3804 (PMC4558179; doi:10.18632/oncotarget.3804)
Supplement: Supplementary file 1 [file oncotarget-06-15681-s001.pdf]

RAS testing in metastatic colorectal cancer: excellent reproducibility amongst 17 Dutch pathology centers

Supplementary Material

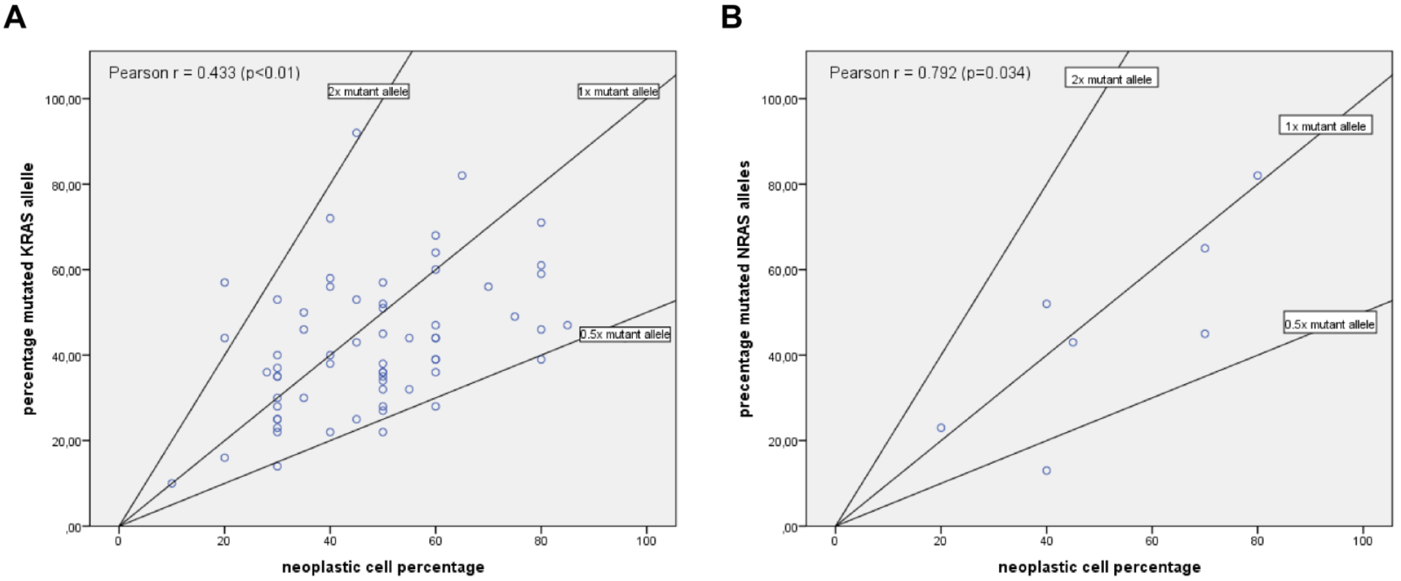

Supplementary Figure 1: The percentage of neoplastic cells in the sample was plotted against the percentage of mutated *KRAS* (**A**) and *NRAS* (**B**) alleles. For both *KRAS* and *NRAS* there is a correlation between the neoplastic cell percentage and the percentage of mutated allelels in the test sample (Pearson r=0.433; p<0.01 and Pearson r=0.792; p=0.034, respectively). In theory, a 100% neoplastic cell content should give 50% mutated alleles when all neoplastic cells in the tumor are heterozygous for the mutation (line 0.5x mutant allele), however, loss of the wild-type allele, copy number gains of the mutated allele and/or inadequate estimation of tumor cell percentage will result in a higher percentage of mutated alleles as illustrated by lines 1x and 2x mutant allele.
